# Reusable and Mediator-Free Cholesterol Biosensor Based on Cholesterol Oxidase Immobilized onto TGA-SAM Modified Smart Bio-Chips

**DOI:** 10.1371/journal.pone.0100327

**Published:** 2014-06-20

**Authors:** Mohammed M. Rahman

**Affiliations:** Chemistry Department & Center of Excellence for Advanced Materials Research (CEAMR), Faculty of Science, King Abdulaziz University, Jeddah, Saudi Arabia; Mathematical Institute, Hungary

## Abstract

A reusable and mediator-free cholesterol biosensor based on cholesterol oxidase (ChOx) was fabricated based on self-assembled monolayer (SAM) of thioglycolic acid (TGA) (covalent enzyme immobilization by dropping method) using bio-chips. Cholesterol was detected with modified bio-chip (Gold/Thioglycolic-acid/Cholesterol-oxidase i.e., Au/TGA/ChOx) by reliable cyclic voltammetric (CV) technique at room conditions. The Au/TGA/ChOx modified bio-chip sensor demonstrates good linearity (1.0 nM to 1.0 mM; *R* = 0.9935), low-detection limit (∼0.42 nM, SNR∼3), and higher sensitivity (∼74.3 µAµM^−1^cm^−2^), lowest-small sample volume (50.0 μL), good stability, and reproducibility. To the best of our knowledge, this is the first statement with a very high sensitivity, low-detection limit, and low-sample volumes are required for cholesterol biosensor using Au/TGA/ChOx-chips assembly. The result of this facile approach was investigated for the biomedical applications for real samples at room conditions with significant assembly (Au/TGA/ChOx) towards the development of selected cholesterol biosensors, which can offer analytical access to a large group of enzymes for wide range of biomedical applications in health-care fields.

## Introduction

Development of cholesterol biosensors in therapeutic diagnostics has gained much attention in health care and biomedical fields. Although cholesterol is essential and important for mammals, higher levels of cholesterol in blood have been linked to damage to arteries and potentially linked to disease such as those associated with the cardiovascular system. With the different experimental parameters, detection of cholesterol in blood sample has considered incredibly significant since its enhancement is related with diabetes, heart diseases, nephrosis, and obstructive jaundice, whereas reduced level of cholesterol is due to mal-absorption wasting syndrome, hypothyroidism, and anemia etc [1]–[4]. Among the various detection techniques of cholesterol, voltammogramic biosensing method has been recently developed as an extremely significant technique [5]. Development of a cholesterol biosensor, immobilization of an enzyme onto self-assembled monolayer fabricated micro-device or bio-chip is usually the primary step in the fabrication of selected biosensor. The selection of an immobilization method is essential for the performance of a biosensor and the future development for fabrication in biosensor design will inevitable focus upon the equipment of innovative devices or chips [6]–[8] that recommend assures to resolve the bio-compatibility and bio-fouling problems. Generally, enzymes are biological catalysts that promote the transformation of chemical species in living systems. These biological molecules, consisting of thousands of atoms in precise arrangements, are able to catalyze the multitude of different chemical reactions occurring in biological living cells. Cholesterol enzymes can catalyze reactions in different states: as individual molecules in solution, in aggregates with other entities, and as attached to fabricated surfaces. The attached-or “immobilized”-state has been of particular interest to those wishing to exploit selective enzymes for practical purposes. The term “immobilized ChOx enzymes” refers to “ChOx enzymes physically confined or localized in a certain defined region of space with retention of their catalytic activities, and which can be used repeatedly and continuously.” As a consequence of ChOx enzyme immobilization, some properties of the enzyme molecule, such as its catalytic activity, stability, become altered with respect to those of its soluble counterpart [9]–[11]. This modification of the properties may be caused either by changes in the intrinsic activity of the immobilized enzyme or by the fact that the interaction between the immobilized selective enzyme and the substrate takes place in a microenvironment that is different from the bulk solution. The observed changes in the catalytic properties upon ChOx immobilization may also result from changes in the three-dimensional conformation of the protein aggravated by the binding of the selective enzyme to the matrix. These effects have been demonstrated and to a lesser extent, exploited for a limited number of enzyme systems [12], [13]. Although the science of enzyme immobilization has developed as a consequence of its technical utility, one should recognize that the advantages of having enzymes attached to surfaces have been exploited by living cells for as long as life has existed. In fact, there is experimental evidence that the immobilized state might be the most common state for enzymes in their natural environment. The attachment of enzymes to the appropriate surface ensures that they stay at the site where their activity is required. This immobilization enhances the concentration at the proper location and it may also protect the enzyme from being destroyed. The term “immobilized enzymes” refers to “enzymes physically confined or localized in a certain defined region of space with retention of their catalytic activities, and which can be used repeatedly and continuously”. Besides the application in industrial processes, the immobilization techniques are the basis for making a number of biotechnological products with applications in diagnostics, bioaffinity chromatography, and biosensors [14]–[16]. The major components of an immobilized ChOx enzyme system are the ChOx, the matrix, and the method of attachment. The ChOx enzymes can be attached to the support by interactions ranging from reversible physical adsorption or ionic linkages to stable covalent bond formation via peptide conjugation. The covalent reactions commonly employed give rise to binding through amide, ether, thio-ether, or carbamate bonds. As a consequence of enzyme immobilization, some properties such as catalytic activity or stability become significantly changed. The concept of stabilization has been an important driving force for immobilizing ChOx enzymes [17]–[19].

High-serum cholesterol is directly related to various health diseases, like arteriosclerosis, heart disease, hypertension, cerebral thrombosis, and coronary artery disease etc [20]–[22]. Hence, the progress of reliable and high sensitive technique for the active and fast detection of cholesterol is an interesting topic recently. It is also enviable to build up a reliable and sensitive cholesterol biosensor, which can allow a suitable and fast detection of cholesterol in blood samples. Various methods have been commenced for the recognition of cholesterol such as, biochemical investigation using radioactive labels [23], [24], HPLC analysis [25], [26], and electrochemical detection [27], [28]. The key drawbacks of these methods are their pitiable sequential, spatial resolutions, and difficulty of the supplementary technical arrangements. Utilization of applicable micro-biosensors could overcome these difficulties with carbon-fiber-based electrodes looking generally efficient [29]–[32]. For conventional methods for biochemical recognition including HPLC on micro-dialysis samples, it was used to immobilize enzyme column and combined electrochemical detectors [33], [34]. A recent study was performed on pH sensitive poly(vinylchloride)membrane with a plasma-polymerized film as a potentiometric biosensor for bio-chemical recognition, where the detection parameters were not satisfactory. However, for this plasma-polymerized film fabricated device, the characteristic curve was not linear, therefore calibration was urgently required. There are a lot of applications for Enzyme Field-Effect Transistors glucose [35], [36], urea [37], [38], acetylcholine [39], [40], and alcohol [41] using various enzymes. The technique of enzyme on biochips is very essential, where experimental immobilization background is concerned on sensitivity and stability. Biosensors based on the principle of field-effect in semiconductor structures have been comprehensively studied in recent years [42]–[44]. Lately, it was potentially developed a charge-transfer-type pH sensor based on a charge-coupled devices [45], , where sensitivity of devices was not achieved in satisfactory-level. With various methodology, the cholesterol biosensors were achieved a substantial interest owing to their sensitivity, selectivity, fast response time, repeatability, and stability. The mediator free electrochemical biosensors are based on suitable immobilization of selective enzyme on proper matrixes offers a portable, economical, disposable, and fast technique for the detection of different bio-molecules. In recent times, researchers are investigated with bio-compatible composite materials as appropriate matrixes for the enzyme immobilization for the efficient recognition of different biological molecules [47], [48]. Among various immobilization techniques, the Au/TGA/ChOx fabricated bio-chips are one of the most promising matrixes which can be used for the immobilization of enzymes due to their numerous interesting properties such as non-toxicity, mediator-less detection, high-surface area, requiring low-sample volume, fast-response time, chemical stability, highly-sensitive, ease of handling, selective, and ease of enzymatic fabrication.

In this report, it is developed highly sensitive mediator-free cholesterol biosensors based on tiny bio-chips at room conditions. Here, it is also measured of the analytical parameters (low sample volume, better sensitivity, and lower-detection limit) of cholesterol biosensor using a bio-chip, which was designed and fabricated successfully by photolithographic method. The most significant characteristics of the developed cholesterol biosensor using TGA/ChOx/bio-chips assembly were highly sensitivity, large-linear dynamic range, very low detection limit, low-sample volume required, selective, highly stable, and reproducible.

## Experimental Sections

### Materials and Methods

**Figure 1:**
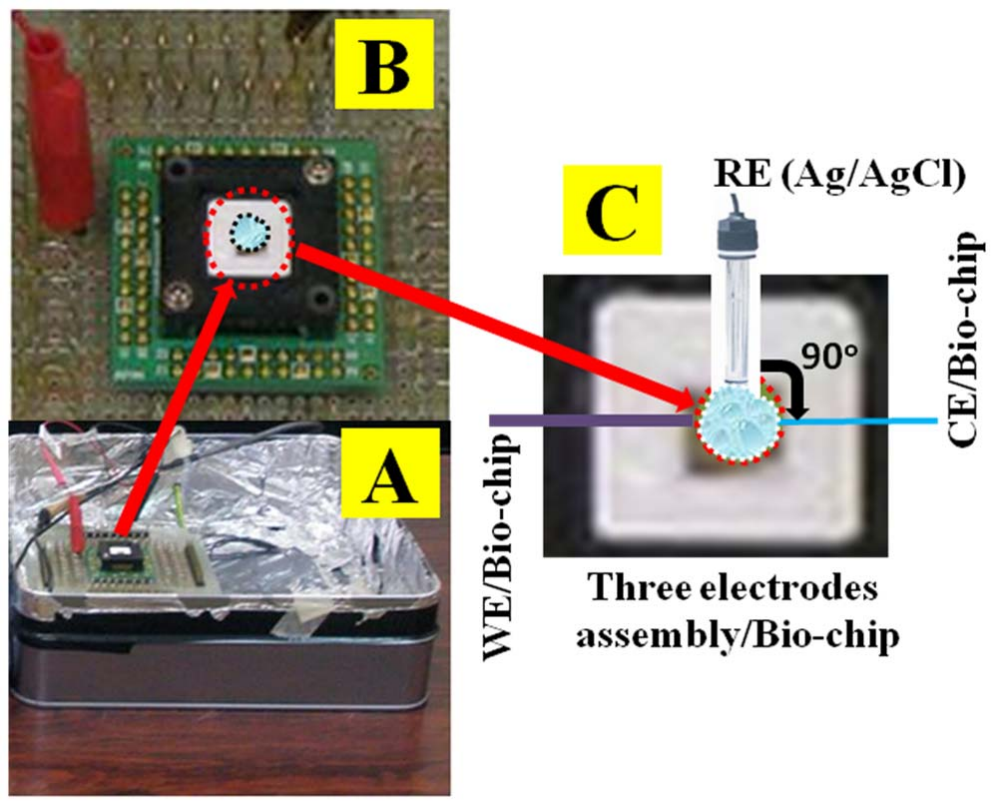
Camera view experimental set-up of (A) potential analyzer (CV-50W) is connected with bio-chip onto electronic board via electronic-pins, (B) magnified view of controlled and fabricated biochip, (C) Three-electrodes system of WE, CE, and RE (perpendicular onto biochip) assembled onto bio-chip. The WE is directly immersed into the electrolyte-drop perpendicularly.

Cholesterol (Ch), N-(3-Dimethylaminopropyl)-N'-ethylcarbodiimide hydrochloride (EDC), thioglycolic acid (TGA), monosodium phosphate (NaH_2_PO_4_), disodium phosphate (Na_2_HPO_4_), and cholesterol Oxidase (ChOx) were obtained from Sigma-Aldrich company (www.sigmaaldrich.com). All other chemicals were analytical-grade and applied without further purification. 0.1 M phosphate buffer solution (pH∼7.1) was prepared by mixing of uni-molar proportion (1∶1) of 0.2 M NaH_2_PO_4_ and 0.2 M Na_2_HPO_4_. Reactants solution was prepared with de-ionized distilled water, which obtained from a water purifying apparatus (12.0 MΩ.cm) (AQUA MEDIA; www.aquamediadirect.com). The electrochemical experiments were measured by using a votammetric-analyzer (CV-50W, BAS, USA; http://www.basinc.com). The experiments were carried out with lab-made bio-chip (5.0 mm×5.0 mm), which micro-level sensing area is 0.0805 cm^2^. The analytical experiments were performed with ChOx enzyme modified bio-chips composed as working, Pt layer as counter, and an Ag/AgCl (saturated KCl) as reference electrodes. Cyclic voltammetry was recorded at AuE/TGA/ChOx/TGA modified electrode from −0.1 to +0.5 V (versus Ag/AgCl) in a 0.1 M phosphate buffer solution (pH 7.1) at 100.0 mV/s scan rates. The total experimental set-up is presented with a camera-view photograph for bio-chips (Fig. 1). Here, the potential analyzer (CV-50W) is controlled and interfaced with SOTECH-PC, which is directly connected with the lab-set electronic system (A) with modified bio-chips (magnified view, B). The three-electrode system including CE, WE, and RE (perpendicular onto biochip) are assembled (C) according to the schematic view in the Figure 1.

### Fabrication of polycrystalline thin-layer of gold onto center of bio-chips

**Figure 2:**
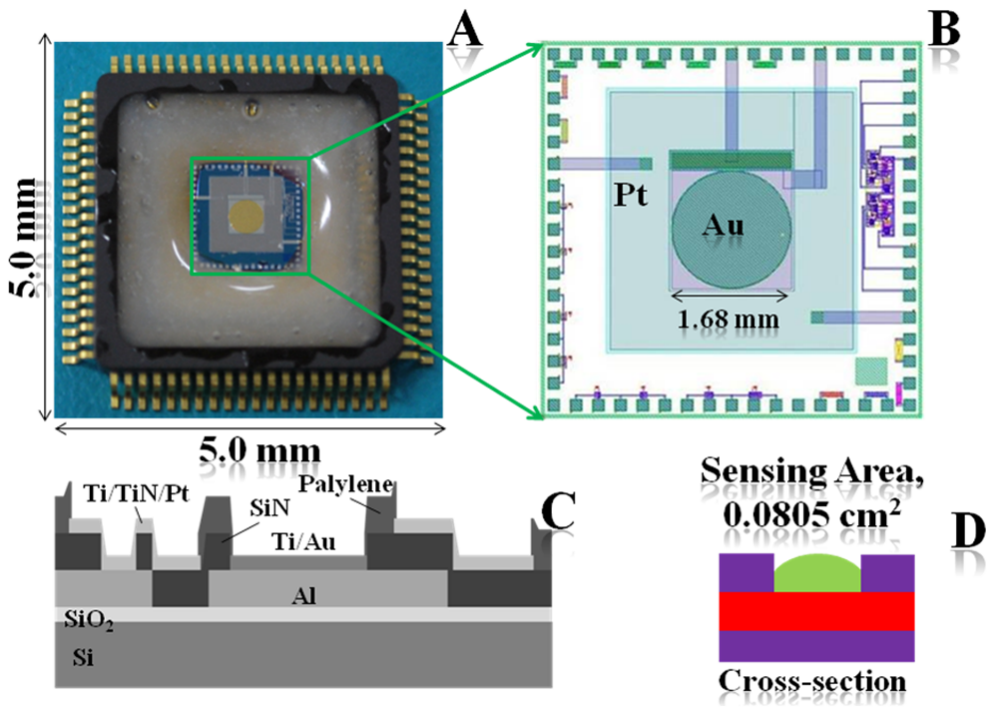
Schematic diagram of (A) top camera-view, (B) magnified view of sensing area, (C) total cross-sectional view, and (D) cross-section of sensing area of bi-chips.

Preparation of biochips by photolithographic method is explained in the ESM section (Ψ). The semiconductor bio-chips were made on silicon wafer. Aluminium was sputtered to fabricate as wiring and bonding pads. Ti/TiN/Pt was sputtered on heated silicon oxide and patterned using photolithography to prepare counter electrode (CE). Ti/TiN layers were used for strong adhesion. Au/Ti were sputtered and lithographed, which prepared circular working-electrode (WE) with a diameter of 1.6 mm in the chip-center. After electrodes fabrication, palylene layer was fabricated using evaporation technique as a passivation layer. The wafer was diced to 5.0×5.0 mm^2^ chips. This chip was bonded to a package by silver paste. Aluminum pads were connected to the package by gold wire. Finally, adhesive (Araldite, Hantsman, Japan) was pasted on the periphery of the biochips to protect the target solution from contacting pads, which is presented in Fig. 2A. The magnified construction view of internal chip-center (sensing area) is shown in the Fig. 2B. A cross section of the total sensor biochip and sensing area is presented in Fig. 2C and Fig. 2D respective.

## Results and Discussion

**Figure 3:**
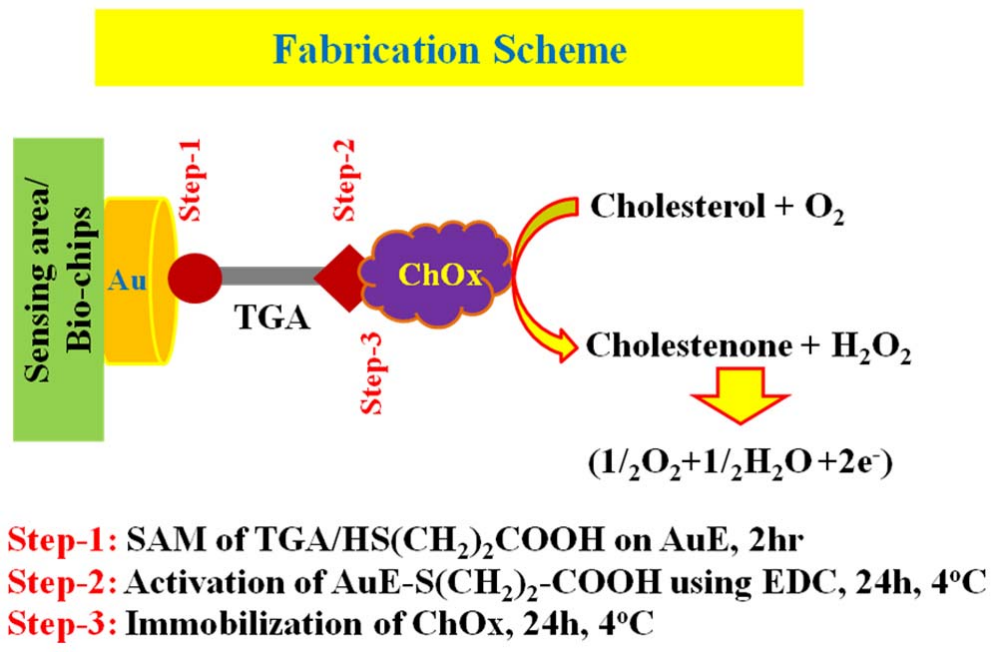
Schematic representation of fabrication procedure for cholesterol biosensor using tiny bio-chips. Sensing area of biochip: 0.0805 cm^2^; TGA: 10.0 mM/ethanol; EDC: 10.0 mM; ChOx: 10.0 mM.

Figure 3 outlines the sensing protocol using the Au/TGA/ChOx-modified bio-chip. It is used for covalent bond formation to immobilize the ChOx enzyme on the TGA-SAM via peptide conjugation in presence of activating agent (EDC). First, the self-assembled monolayer of TGA is formed by dropping the ethanolic solution of TGA onto bio-chips for two hours. Then ChOx enzyme is immobilized onto TGA-SAM electrode by amide-bond formation between the terminal-unbound carboxylic acids (-COOH) group of TGA SAM and the amine groups (-NH_2_) of ChOx enzymes.

H_2_O_2_ is a member of the reactive oxygen species that play many important roles in biology and medicine, including immune response, ageing, cell signaling, and wound healing. The major source of H_2_O_2_ in mammalian cytosols is the leakage from the mitochondrial electron transportation chain. This leakage generates superoxide anion radical (O_2_−•). The generated superoxide anion can be rapidly transformed into H_2_O_2_ by superoxide dismutase activity. Also, H_2_O_2_ can be generated by activity of oxidases, after which it participates in various physiological events [49]. Nanotechnology can provide possible working solutions to overcome these drawbacks and promise more accurate and precise means to investigate H_2_O_2_'s action in various biological events. For the stable attachment of ChOx onto TGA-SAM, the chip was kept for 24 hours into the refrigerator at 4.0°C. The enzymatic reactions were performed on the bio-sensing system in biochips for the efficient detection of cholesterol as follows (see Fig. 3):
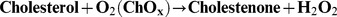
(1)

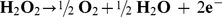
(2)


Reaction (1) is depended on cholesterol concentration in the reaction medium. On bio-chip, cholesterol is oxidized to form cholestenone and H_2_O_2_ in presence of ChOx. Then H_2_O_2_ is auto-dissociated to produce the free-electrons/current (Reaction 2). This current is directly proportional to the injected cholesterol concentration in the solution system. H_2_O_2_ is an important member of the reactive oxygen species, playing various roles in biology and medicine. The conventional detection methods for H_2_O_2_ are often restricted by their limited sensitivity, good selectivity towards H_2_O_2_, appropriate physicochemical properties for detection in biological environments, short response time, etc [50]. Here, H_2_O_2_ is an electroactive molecule produced as byproduct by the enzymatic reaction cholesterol in presence of ChOx. After formation of H_2_O_2_, it is auto-dissociated on the electrode surface of an electrode, generating an electric current. In order to improve current electrochemical sensing techniques, research interests in H_2_O_2_ detection have been focused on surface modification, so as to improve sensor performance. By modifying or architecting the electrode surface, using appropriate electrode assembly with SAM modification or nanostructures materials, various advantages have been obtained, such as large surface area for sensing, improved electric conductivity, ability to accumulate analyte, surface functionalization, and electro-catalytic activity [51].

**Figure 4:**
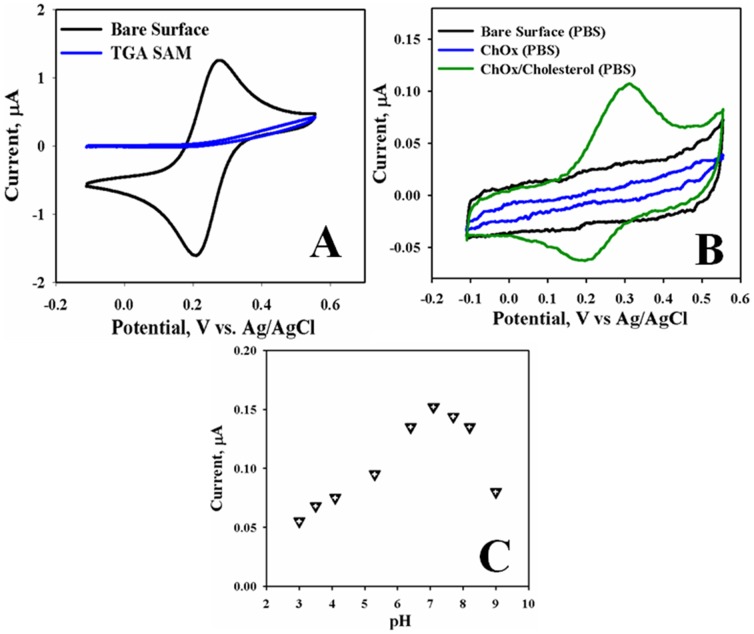
(A) CVs of 5.0 mM K_3_Fe(CN)_6_/PBS (0.1 M) for bare (black curve) and TGA-SAM (blue curve) modified electrodes; (B) CVs recorded in 0.1 M phosphate buffer solution of bare chip (black curve), ChOx modified chip (blue curve), and in presence of 0.1 mM cholesterol (green curve) solution; (C) pH effect of Au/TGA/ChOx electrode in 0.1 mM cholesterol solution with the bio-chip. Scan rate: 0.1 V/s, RE: Ag/AgCl (saturated KCl), Supporting electrolytes: 0.1 M phosphate buffer solution.

Successful fabrication of cholesterol sensor using ChOx on the sensing area of bio-chip was confirmed based on TGA-SAMs by cyclic voltammetric techniques. Conventional electrochemical method is the most versatile electroanalytical technique for the study with bio-active materials and species, which was extensively used in industrial applications and academic research for R & D approaches. CV is also a significant method to assess the blocking property of the monolayer-coated electrodes using diffusion controlled redox couples. Chip surface was cleaned by Piranha solution [H_2_SO_4_∶H_2_O_2_ (3∶1)] and washed with pure water, then dried adequately by nitrogen. TGA was dissolved in ethanol to make 10.0 mM solution. TGA solution was dropped on a sensing area of bio-chip, and then kept wet for two hours at room conditions. Fig. 4 shows the CVs of un-modified and TGA-SAM modified bio-chip electrodes in 5.0 mM K_3_Fe(CN)_6_ with 0.1 M PBS as the supporting electrolyte at 0.1 V/s scan rates. It can be seen from the Fig. 4A, that the bio-chip electrode (black-curve) shows a reversible voltammogram for the redox couple indicating that the electron transfer reaction is completely diffusion controlled. In contrast, the absence of any peak formation in the CVs of the TGA monolayer modified electrodes (blue-curve) shows the redox reaction is inhibited or totally blocked. The CVs for TGA signified a good blocking behavior for the electron transfer reaction, which means that a highly ordered, compact monolayer is formed on the sensing surface of the bio-chips [52].

Enzyme (ChOx) was embedded onto the TGA-SAM modified surface by the phenomena of peptide conjugation. First 10.0 mM EDC in 0.1 M phosphate buffer solution was put onto the TGA-SAM bio-chip and then it was kept at 4.0 °C in the refrigerator to activate carboxylic group of TGA for 24 hours. Then EDC-treated electrode was washed slowly with 0.1 M PBS to eliminate excess EDC. Then ChOx solution was dropped onto the sensing area (center) of bio-chip and incubated in the refrigerator at 4.0°C for 24 hours. ChOx was effectively immobilized onto TGA SAM via covalent attachment, which was confirmed by the current change in Fig. 4B. It showed that the CVs recorded for the bare-surface (black curve), Au/TGA/ChOx (blue curve), and 0.1 mM cholesterol solution (green curve) of fabricated bio-chips in a 0.1 M PBS at 0.1 V/s scan rates. According to the control experiment, no significant change was observed when the CV was recorded with the bare-surface for 0.1 mM cholesterol in phosphate buffer solution owing to the absence of ChOx enzyme. A small current change was observed at approximately +0.32V versus Ag/AgCl (sat. KCl) for 0.1 mM cholesterol solution and this was due to the current of enzymatic reaction with cholesterol in presence of ChOx on the sensing surface of the bio-chips. The enzymatic current approximately +0.32 V was executed to be increased on the increasing cholesterol concentration in the PBS buffer solution. The experimental conditions affecting the performances (detection limit, sensitivity, and response time) of the biosensors were optimized in term of pH and presented in Fig. 4C. The pH of the buffer shows a strong effect on the activity of the sensing layer on ChOx enzyme fabricated bio-chips. The effect of pH on the current change is studied in the phosphate buffer system in pH range of 3.0 to 9.1. Fig. 4C shows the peak currents measured (in CV) at various pH values for 0.1 mM cholesterol in 0.1 M phosphate buffer solution system. The peak height is increased from pH 5.6 to 7.1 and then decreased above pH 7.1 until 9.1. The peak current decreases above pH 7.1, which might have been owing to the poor ChOx enzyme activity at higher pH medium. Therefore, the pH of the phosphate buffer solution system is kept constant at 7.1 throughout the investigation.

**Figure 5:**
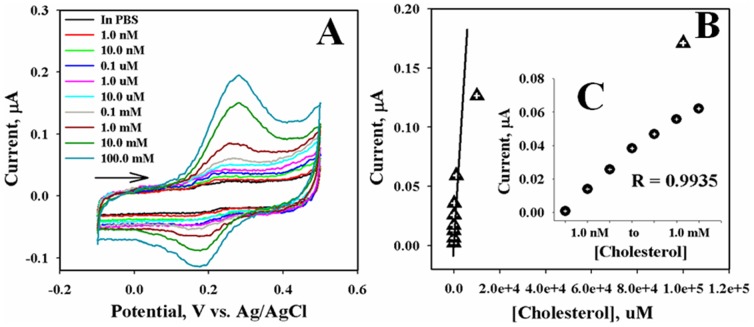
Electrochemical sensor responses of (A) variation of cholesterol concentrations (1.0 nM to 100.0 mM), (B) calibration curve, and (C) linearity of developed on bio-chip at room conditions. Scan rate: 0.1 V/s; Concentration range of cholesterol: 1.0 nM to 100.0 mM; Method: CV; Reference electrode: Ag/AgCl (sat. KCl).

Cyclic voltammetric was performed to validate the detection of cholesterol concentration without mediators in simple phosphate buffer solution system. 50.0 μL of each cholesterol solution without mediator was dropped on the sensing area of bio-chips and measured the sensing oxidation current. Fig. 5A exhibits a typical CV (current-voltage) plot for the addition of varying amounts of target cholesterol in a 0.1 M phosphate buffer solution (pH 7.1). The current is increased gradually with increasing the concentration of cholesterol (1.0 nM to 100.0 mM) towards stable and saturated value. The Au/TGA/ChOx modified bio-chip electrode is achieved 96.0% of steady state currents with in 10.0 sec. The increase of oxidation current is presented due to the cholesterol oxidized in presence of ChOx and the current change leads to the higher current value. Fig. 5B shows the calibration plots for the cholesterol found from the current-voltage responses with fabricated bio-chips. Under the optimized conditions, the steady-state currents are exhibited a linear relationship with the target cholesterol concentration in the range from 0.1 nM to 1.0 mM, which is presented in Fig. 5C.

The linear dependence of the cholesterol concentration acquiesced with a correlation coefficient of 0.9935. The detection limit for cholesterol was executed to be approximately 0.42 nM, based on signal to noise ratio (^3N^/_S_). The cholesterol biosensor also exhibited with higher sensitivity, which was calculated as **7**4.3±0.5 µA.µM^−1^.cm^−2^. The sensitivity is much higher than the previously reported cholesterol biosensors, where the total comparison is included in Table 1. For comparing, the biosensor performances of ChOx fabricated bio-chip is compared with the previously reported cholesterol biosensors based on the enzymes conjugated various materials and confirmed that the Au/TGA/ChOx-biochip cholesterol biosensor performances exhibited excellent results.

**Table 1:** Evaluation of the Au/TGA/ChOx-biochip cholesterol sensor performances compared with various enzyme-material conjugated sensors.

Electrode materials	Sensitivity	Detection limit	Sample volume (μL)	Response time (s)	Linear dynamic range (LDR)	Linearity (R)	References
**ZnO nanoparticles +chitosan composite**	14.1 µAµM^-1^cm^−2^	0.125×10^3^ µM	—	15	(0.125–7.76)×10^6^ µM	-	[53]
**ZnO nanoporous thin film**	—	—	—	15	(0.65–10.35)×10^6^ µM	—	[54]
**Tertraethylorthosilicate**	—	0.50 µM	—	50	(2.0–10.0)×10^3^ µM	—	[55]
**ZnO nanorods**	61.7 µAµM^−1^cm^-2^	0.012 µM	—	5	1.0–15.0 µM	0.9979	[56]
**Polypyrrole films**	15.0 µAµM^−1^cm^−2^	—	—	—	1.0–8.0×10^3^ µM	—	[57]
**ChOx/NanoPt/CNT**	—	—	—	<20	4.0×10^−6^–1.0×10^−4^ mol L^−1^	—	[58]
**ZnO nanoparticles**	23.7 µAµM^−1^cm^-2^	0.37 mM	—	5	0.001–0.5 µM	—	[59]
**Nanoporous CeO_2_ film**	5.98 µAµM^−1^cm^−2^	—	—	15	1.3–10.35×10^6^ µM	—	[60]
**ChOx/CS/MWCNT**	1.55 µAmM^−1^	**—**	**—**	<20	4.0×10^−6^ to 7.0×10^−4^	**—**	[61]
**Ni/K3Fe(CN)6/CNT**	**—**	**—**	**—**	<20	0.005–3 mM	**—**	[62]
**ChOx/NanoZnO-CHIT**	1.41×10^−4^ AmgdL^−1^	**—**	**—**	**15**	05–300 mgdL^−1^	**—**	[63]
ChOx/4-ATP/Au	542.3 nA mM^−1^	**—**	**—**	**20**	25 to 400 mg dl^−1^	**—**	[64]
**Au/TGA/ChOx-biochip**	**74.3137 µAµM^−1^cm^−2^**	**0.42 nM**	**50.0**	**10**	**1.0 nM–1.0 mM**	**0.9935**	**Current work**

A series of successive measurement of cholesterol in 0.1 M phosphate buffer solution yielded a good reproducibility signal at Au/TGA/ChOx biosensor with a relative standard deviation (RSD) 3.2%. The sensor-to-sensor and run-to-run reproducibility for 0.1 mM cholesterol detection were found to be 1.5 and 1.1% respectively. To study the long-term storage stabilities, the response of the Au/TGA/ChOx sensor was done with the respect to the storage time. After each experiment, the sensor was washed with the buffer solution and stored in 0.1 M phosphate buffer at 4.0°C until use. The long-term storage stability of the sensor was tested every three days. The sensitivity retained 87% of initial sensitivity up to one month. After one month, the response slowly decreased, probably due to the loss of the enzyme activity on the sensor bio-chip. The above results clearly indicated that the bio-chip of cholesterol sensor can be used for a month without any significant loss in sensitivity. The selectivity [interference effect, presented in ESM (Φ)] of the Au/TGA/ChOx sensor was evaluated in the presence of other electro-active compounds such as lactate, glucose, ascorbic acid, glutamate, and uric acid etc. No significant current-response was found when 0.1 mM lactate, glucose, uric acids, ascorbic acid, and glutamate were injected into the 0.1 M phosphate buffer solution buffer system. But when 0.1 mM cholesterol solution was added to the electrolyte solution, a clear oxidation response was observed, indicating the selective detection of cholesterol with the Au/TGA/ChOx sensor layer. Additionally, at this concentration level (lactate, glucose, uric acid, and glutamate), these were not showed any interfere in 0.1 mM cholesterol detection using bio-chips. Thus, the selectivity of the Au/TGA/ChOx bio-chip sensor is acceptable for cholesterol detection in the presence of the common interfering compounds in normal physiological levels. In order to check the accuracy of the developed methods, analytical recovery of added cholesterol in five real serum samples was investigated. The mean analytical recovery of added cholesterol (∼2.76 mM) in serum was 92.1±1.5% (n = 5), which was higher than pencil graphite rod electrode by amperometric method [65]. The simple fabrication method of the biosensor has several advantages over conventional technique such as ease of fabrication, enhanced electro-catalysis, and efficiently preserving the activity of bio-molecules.

## Conclusions

Successful fabrication of highly sensitive cholesterol biosensor based on ChOx immobilization onto gold-thin layer fabricated sensor bio-chip has been investigated in mediator-free enzymatic system. Sensor bio-chips were constructed by photolithographic technique using polycrystalline gold-thin layers, where it was possible to detect the target cholesterol solution with selective ChOx enzyme in tiny micro-volume (∼50.0 μL). Cholesterol biosensors were exhibited higher sensitivity (∼74.3 µAµM^−1^cm^−2^), low-detection limit (∼0.42 nM) with satisfactory stability, large linear dynamic range (1.0 nM to 1.0 mM), and reproducibility. This mediator-free cholesterol biosensor using bio-chip can be used to estimate cholesterol in micro-level quantity. It would have potential applications in cholesterol determination in health care biological and biomedical fields. Finally, this is the first report in which such a high-sensitivity and low-detection-limit have been introduced with ultra-sample volume for the mediator-free cholesterol detection by Au/TGA/ChOx bio-chips assembly at room conditions.

## Supporting Information

File S1 **Graphical Abstract.** Fabrication of highly sensitive cholesterol biosensor based on ChOx immobilized Thioglycolic acid (TGA) conjugated self-assembled monolayer (SAM) onto smart bio-chips.
(DOCX)
