# Supplementary material for: Reusable and Mediator-Free Cholesterol Biosensor Based on Cholesterol Oxidase Immobilized onto TGA-SAM Modified Smart Bio-Chips
Source: PLoS One. 2014 Jun 20;9(6):e100327. doi: 10.1371/journal.pone.0100327 (PMC4065056; doi:10.1371/journal.pone.0100327)
Supplement: File S1 — Graphical Abstract. Fabrication of highly sensitive cholesterol biosensor based on ChOx immobilized Thioglycolic acid (TGA) conjugated self-assembled monolayer (SAM) onto smart bio-chips. (DOCX) [file pone.0100327.s001.docx]

**Reusable and mediator-free cholesterol biosensor based on cholesterol Oxidase immobilized onto TGA-SAM modified smart bio-chips**

**Mohammed M. Rahman***

**…………………………………………………………………………**

**Graphical Abstract**

Fabrication of highly sensitive cholesterol biosensor based on ChOx immobilized Thioglycolic acid (TGA) conjugated self-assembled monolayer (SAM) onto smart bio-chips.

**Electronic Supplementary Materials (ESM):**

(Ψ) Preparation of chips by photolithographic method:

Electrochemical chips were constructed by conventional photolithographic technique, where electrodes and passivation layers of sensor are fabricated on silicon wafer followed by dicing and packaging. Nitrogen-doped Silicon wafers are prepared and overflowed by extra-pure de-ionized water. Here, the contaminants on the surface as well as native SiO2 layer are removed. At first, the wet oxidation technique is processed and then dry oxidation is executed. Wafers are annealed in the condition of nitrogen system, and then Aluminum is sputtered with Al-1% Si target. After that the photolithograph processes are applied. By Kanto chemicals, resist coating, baking, exposure, and development are executed, and then it is rinsed thoroughly by ionic water. Aluminium is etched by etching solution. Resist is removed by plasma etching instrument. Then wafers are cleaned by acetone, methanol, and finally by plasma simultaneously. Silicon nitride layer is deposited by chemical vapor deposition. Surface of pad electrodes are etched by reactive ion etching. Finally residual resist layer is removed by plasma acing. After photolithographic process, platinum (Pt) is sputtered using SP150-HTS. Then it is patterned by lift-off method, in which wafers are fully-immersed into the remover solution. Later it is washed with Isopropyl alcohol (IPA) carefully. Photolithographic process is repeated again, where titanium (Ti) is sputtered here as a binding-layer, and then gold (Au) is evaporated by deposition technique. Finally, gold layer is patterned by lift-off procedure. Palylene passivation layer is made for the protection of chip using water. Photolithographic technique is carried out again for pad protection. Then palylene-dimer is evaporated by deposition apparatus. Photolithography process is made again for patterning. Palylene layer is patterned by etching. Finally, the unnecessary resists are removed successfully by acetone and then wafer is cleaned perfectly by IPA. Resist is coated on the whole surface of the wafer for protection during dicing method. Si wafer is diced into pieces by dicing apparatus and stored into the desiccators. Resist on chip surface is removed by acetone and cleaned with IPA. The backside of the chip is roughed by a sheet of sandpaper for better adhesion and electrical stability. The chip is bonded with die and packaged by silver paste. It is dried in a drying oven. Pads on chip are connected to the package through gold wire with bonding machine. Finally, Si-based adhesive is put on the periphery of the chip to protect pads and gold wire from sample solution. Adhesive is dried at room temperature for 24 hours. The composition with thickness of each fabricated layer into bio-chips are Si wafer material (500.00 µm), SiO_2_ insulation (0.40 µm), Al electric wiring (1.00 µm), SiN protection/separation (1.00 µm), Ti/TiN Binding (0.15 µm), Ti binding (0.10 µm), Pt counter electrode (0.25 µm), Au working electrode (0.30 µm), and Palylene passivation/protection (1.00 µm).

**(Φ) Interferences study:**

The selectivity of the Au/TGA/ChOx sensor was evaluated in the presence of lactate, glucose, ascorbic acid, glutamate, and uric acid and presented in below (Figure S1).
